# Vaccination coverage according to immunosuppression level among adults with non-rheumatoid arthritis rheumatic diseases: a retrospective observational study in Korea

**DOI:** 10.4178/epih.e2026018

**Published:** 2026-04-23

**Authors:** Choseok Yoon, Kiho Jeong, Wooyoung Jang, Jinnam Kim, Se Yoon Park, Bongyoung Kim

**Affiliations:** 1 Division of Infectious Diseases, Department of Internal Medicine, Hanyang University Seoul Hospital, Seoul, Korea; 2 School of Medicine, Hanyang University College of Medicine, Seoul, Korea; 3 Department of Internal Medicine, Hanyang University College of Medicine, Seoul, Korea

**Keywords:** Autoimmune diseases, Rheumatic diseases, Vaccine-preventable diseases, Vaccination, Vaccination coverage, Immunosuppression therapy

## Abstract

Patients with autoimmune inflammatory rheumatic diseases are particularly vulnerable to infectious diseases. Accordingly, a broader range of vaccines and, in some cases, additional doses are recommended for these patients compared with the general population. This study investigated the vaccination status of adult patients with non-rheumatoid arthritis rheumatic diseases (NRRDs). This retrospective study was conducted in the rheumatology outpatient department of a tertiary care hospital and included adult patients (aged ≥19 years) with NRRDs who received glucocorticoids and/or disease-modifying antirheumatic drugs from January 2023 to March 2023. Among these patients, we compared vaccination rates between high-level immunosuppression (HLI) and non-HLI groups. HLI was defined as current biologic use or prednisolone equivalent at a dose of ≥20 mg/day for ≥14 days. Of the 4,070 patients with NRRDs, 1,522 (37.4%) were in the HLI group. Overall vaccination rates (complete plus partial vaccination) were significantly lower in the HLI group than in the non-HLI group for influenza (20.2 vs. 31.0%, p<0.001), hepatitis B (59.5 vs. 68.8%, p<0.001), pneumococcal disease (12.9 vs. 26.9%, p<0.001), and herpes zoster (4.3 vs. 10.8%, p<0.001). Overall, vaccination rates were low among patients with NRRDs, with especially low rates in the HLI group. These findings highlight the need for systematic vaccination strategies.

## GRAPHICAL ABSTRACT

[Fig f2-epih-48-e2026018]

## Key Message

Despite being at high risk for infection, most adult patients with non-rheumatoid arthritis rheumatic diseases (NRRDs) did not complete the recommended adult vaccinations. Vaccination rates, including those for the influenza, pneumococcal, and herpes zoster vaccines, were significantly lower in the high-level immunosuppression (HLI) group than in the non-HLI group. Overall, these findings reveal a critical gap in the preventive care of immunocompromised patients with NRRDs and underscore the need for systematic vaccination strategies in this vulnerable population.

## INTRODUCTION

Vaccination is one of the greatest achievements in the history of public health. In the United States, immunization programs have substantially reduced the incidence and mortality of many vaccine-preventable diseases (VPDs) [1]. Patients with autoimmune inflammatory rheumatic diseases (AIIRDs) are more vulnerable to infectious diseases, including VPDs, than the general population due to the underlying disease and immunosuppressive medications, such as glucocorticoids, disease-modifying antirheumatic drugs (DMARDs), conventional synthetic DMARDs (csDMARDs), biologic DMARDs (bDMARDs), and targeted synthetic DMARDs [2-5]. Several studies have estimated that the incidence of severe infections in these patients is approximately twice as high as that in the general population [6,7].

To mitigate this risk, major international societies, including the European Alliance of Associations for Rheumatology, the American College of Rheumatology, the Korean College of Rheumatology, and the Korean Society of Infectious Diseases (KSID), strongly recommend appropriate vaccinations for patients with AIIRDs [2,3,8,9]. Vaccination is one of the most cost-effective and clinically effective strategies for reducing the incidence and severity of infectious diseases in immunocompromised patients [3,10]. Despite these clear recommendations, several studies have shown that vaccination rates in patients with AIIRDs remain very low, often lower than those in the general population [10,11].

Most previous studies have focused on relatively common rheumatic diseases, such as rheumatoid arthritis, while few have examined real-world vaccination status in patients with non-rheumatoid arthritis rheumatic diseases (NRRDs), a diverse and understudied subset of AIIRDs. Additionally, the degree of immunosuppression is a key determinant of both infection risk and vaccine-related decision-making in clinical practice. However, it remains unclear whether real-world vaccination coverage differs according to the degree of immunosuppression in patients with NRRDs.

For these reasons, this study aimed to evaluate vaccination status among patients with NRRDs according to the degree of immunosuppression by comparing high-level immunosuppression (HLI) and non-HLI groups.

## MATERIALS AND METHODS

### Data source

**Figure 1.**
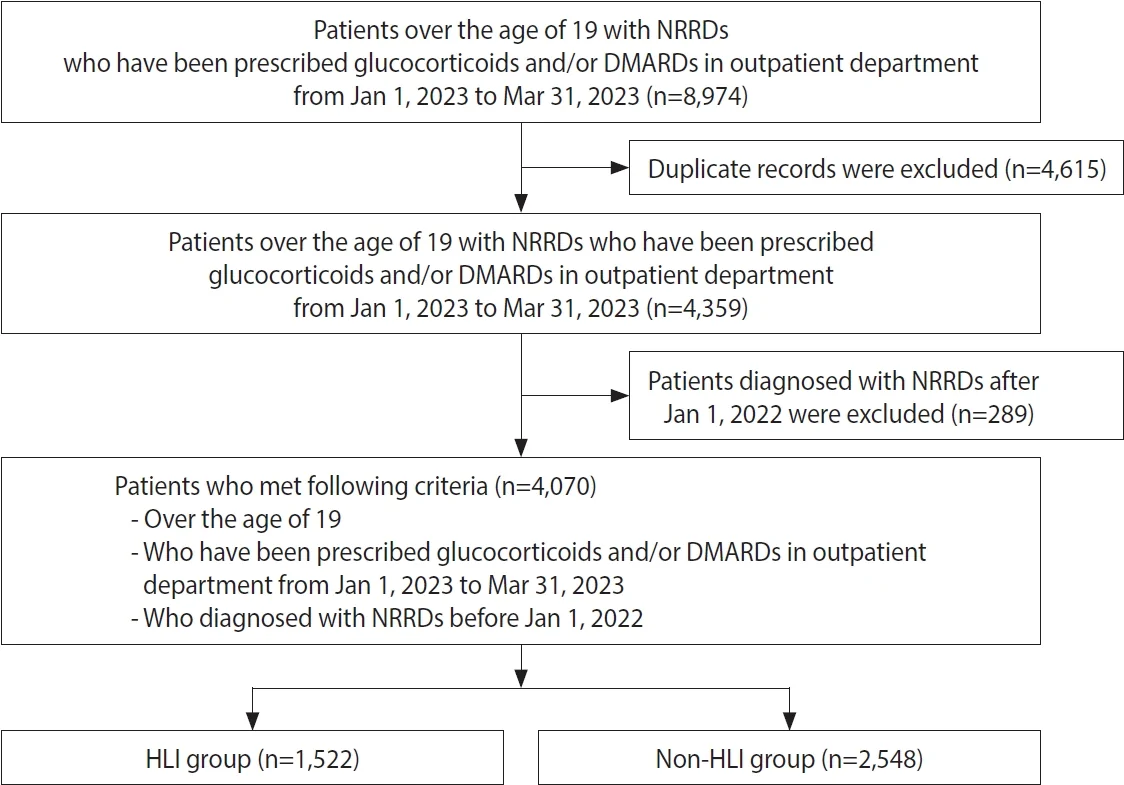
Flowchart of the patient selection process. This figure presents the patient selection process for the HLI and non-HLI groups. NRRDs, non-rheumatoid arthritis rheumatic diseases; DMARDs, disease-modifying antirheumatic drugs; HLI, high-level immunosuppression.

This retrospective single-center study was conducted at a tertiary care hospital, the largest independent rheumatology center in Korea, from January 2023 to March 2023. We analyzed all adult patients (aged ≥19 years) with NRRDs, as defined according to the Copayment Decreasing Policy of Korea’s National Health Insurance System, who received DMARDs in the rheumatology outpatient department during the study period. Patients diagnosed with NRRDs after January 1, 2022, were excluded because there was insufficient time to assess their vaccination history. A flowchart of the patient selection process is shown in Figure 1, and the types of NRRDs included in this study are detailed in Supplementary Material 1.

Vaccination information about the enrolled patients was collected by reviewing both electronic health records (EHRs) and the vaccination management system of the Korea Disease Control and Prevention Agency.

### Study groups and definitions

Patients were classified into the HLI and non-HLI groups based on the degree of immunosuppression. The HLI group was defined as including: (1) patients who were prescribed biologics, (2) patients who were prescribed prednisolone or an equivalent at a dose of ≥20 mg/day for more than 14 days, (3) patients who had undergone solid organ transplantation within 2 months, and (4) patients who had undergone hematopoietic stem cell transplantation within 2 years [6,12,13]. A list of the VPDs was obtained from the 2019 KSID Vaccination Guidelines for Adults [8,14] (Supplementary Material 2). The types of DMARDs used by patients in this study are listed in Supplementary Material 3 [2,15].

The category “not indicated” includes patients for whom vaccination was not indicated or could not be classified due to insufficient information. More specifically, “not indicated” was defined as follows: for hepatitis A virus (HAV), individuals who were anti-HAV antibody-positive with no history of HAV vaccination, or individuals aged ≥40 years with no history of HAV vaccination and no available laboratory results for anti-HAV antibodies; for hepatitis B virus (HBV), individuals who were HBV carriers; for herpes zoster, individuals aged <50 years who had received a live zoster vaccine; and for human papillomavirus (HPV), individuals aged >26 years.

For HAV, because a substantial proportion of patients were categorized as “not indicated,” statistical comparisons were performed in the population with known status after excluding the remaining patients from the denominator. Supplementary age-stratified analyses were also conducted to explore the potential influence of age-related HAV seropositivity and unavailable antibody results.

### Statistical analysis

All statistical analyses were performed using SPSS version 21 (IBM Corp., Armonk, NY, USA). Continuous variables were compared using the independent *t*-test, and categorical variables were compared using the chi-square test.

For each vaccination, we compared the combined proportion of patients who were completely or partially vaccinated between the HLI and non-HLI groups. In addition, to determine whether the level of immunosuppression independently predicted low vaccination coverage, we performed multivariable logistic regression analysis after adjustment for age and sex.

A two-tailed p-value <0.05 was considered to indicate statistical significance.

### Ethics statement

The study protocol was approved by the Institutional Review Board of Hanyang University Seoul Hospital (approval No. 2023-09-11).

## RESULTS

### Demographic characteristics of the high-level immunosuppression (HLI) and non-HLI groups

A total of 4,070 patients with NRRDs were included in the study, of whom 1,522 (37.4%) were classified in the HLI group. The mean age was 42.6±12.4 years in the HLI group and 47.0±14.2 years in the non-HLI group, a statistically significant difference (p<0.001). The proportion of female patients also differed significantly between the groups (p<0.001): 27.5% in the HLI group and 86.1% in the non-HLI group. Ankylosing spondylitis (87.6 vs. 9.3%, p<0.001) and systemic lupus erythematosus (5.3 vs. 59.2%, p<0.001) were the most common rheumatic diseases in the HLI and non-HLI groups, respectively, with both differing significantly in frequency between the groups (Table 1).

### Types of medications in the high-level immunosuppression (HLI) and non-HLI groups

Adalimumab (32.0%) was the most frequently used medication in the HLI group, followed by etanercept (30.2%), infliximab (17.7%), and golimumab (12.0%). In contrast, hydroxychloroquine (71.6%) was the most commonly used medication in the non-HLI group, followed by low-dose corticosteroids (65.9%), methotrexate (21.9%), and sulfasalazine (10.2%) (Table 2).

### Vaccination status of the high-level immunosuppression (HLI) and non-HLI groups

Regarding the recommended adult vaccines, 27.0% of patients had completed influenza vaccination, and this proportion was significantly lower in the HLI group than in the non-HLI group (20.2 vs. 31.0%, p<0.001). For hepatitis A vaccination, 10.9% of patients had received at least 1 dose, with no significant difference between groups (11.3 vs. 10.5%, p=0.535). For hepatitis B vaccination, 65.3% of patients had received at least 1 dose, and the proportion was significantly lower in the HLI group than in the non-HLI group (59.5 vs. 68.8%, p<0.001). In addition, 21.6% of patients had received pneumococcal vaccination (13-valent pneumococcal conjugate vaccine [PCV13] or 23-valent pneumococcal polysaccharide vaccine [PPSV23]), and this proportion was significantly lower in the HLI group than in the non-HLI group (12.9 vs. 26.9%, p<0.001). Furthermore, 8.3% of patients had received herpes zoster vaccination (live or inactivated vaccine), with this proportion significantly lower in the HLI group (4.3 vs. 10.8%, p<0.001). Regarding HPV vaccination, 1.5% of patients had received at least 1 dose, and this proportion was also significantly lower in the HLI group (0.4 vs. 2.2%, p<0.001) (Table 3).

Because the sex distribution differed substantially between the HLI and non-HLI groups, additional sex-stratified analyses were performed separately for male and female patients (Supplementary Materials 4 and 5). Even after stratification by sex, differences in vaccination rates for the major VPDs remained significant between the 2 groups, except for HPV.

In addition, for certain VPDs for which recommended vaccination schedules may differ by age group (HAV, pneumococcal disease, and herpes zoster), we stratified patients according to the relevant age cutoffs and reanalyzed the data accordingly (Supplementary Materials 6-8).

### Multivariable logistic regression of factors associated with vaccination coverage

After adjustment for age and sex, multivariable analysis showed that HLI was independently associated with lower vaccination coverage for HBV (odds ratio [OR], 0.79; 95% confidence interval [CI], 0.65 to 0.91; p=0.002), tetanus/diphtheria/acellular pertussis (Tdap)/tetanus/diphtheria (Td) (OR, 0.76; 95% CI, 0.60 to 0.96; p=0.019), and pneumococcal vaccination (OR, 0.71; 95% CI, 0.57 to 0.89; p=0.003) (Table 4).

## DISCUSSION

To our knowledge, this is the first real-world study in Korea to investigate vaccination status in patients with NRRDs according to the degree of immunosuppression. Patients in the HLI group were younger and more likely to be male, which may reflect the high prevalence of ankylosing spondylitis, a male-predominant condition, in this group [16,17]. In contrast, diseases such as systemic lupus erythematosus and systemic sclerosis, which are more prevalent in women, were more common in the non-HLI group [18,19]. These disease distributions likely reflect real-world treatment patterns in which bDMARDs are prescribed more frequently for certain inflammatory conditions, resulting in a higher proportion of patients with HLI. In fact, bDMARDs exert stronger immunosuppressive effects than csDMARDs because they directly inhibit specific immune pathways. Several studies have reported higher rates of infection and hospitalization among patients treated with bDMARDs than among those receiving csDMARDs [20-22].

Our findings suggest a substantial gap between clinical guidelines and real-world practice. This highlights a notable paradox in the care of patients with NRRDs: despite their increased vulnerability to infectious diseases due to immunosuppressive therapy, vaccination uptake in this population, particularly among those in the HLI group, remained low across nearly all recommended vaccines, including influenza, pneumococcal, and herpes zoster vaccination. These findings may be explained by factors at both the patient and healthcare provider levels, including limited patient awareness, concerns about vaccination, and insufficient clinician recognition of the importance of vaccination [10,23]. In a single-center survey-based study, 14.7% of patients with rheumatic diseases reported that they had never been advised to receive pneumococcal vaccination, while 8.2% indicated that influenza vaccination had never been recommended [23]. Additionally, concerns about potential disease flare-ups or vaccine safety may contribute to low vaccination rates [24,25]. However, multiple large-scale studies have found no significant increase in disease activity following most recommended vaccines [26,27].

Several strategies have been proposed to improve vaccination rates in this population, including computerized alert systems, financial support for vaccination, patient education, and standardized vaccination policies [11]. In a study in the United States, strategies such as reminders and recalls, onsite vaccination, and behaviorally targeted incentives were effective in increasing vaccination uptake [25]. Extending these strategies to adult populations, particularly immunocompromised patients such as those with AIIRDs, may improve vaccination uptake in these high-risk groups.

This study had several limitations. First, because this was a single-center study, its findings may not be fully generalizable to the national population. Second, the accuracy and completeness of data extracted from EHRs may have been limited because retrospective chart reviews are inherently subject to documentation errors and missing information. In addition, some patients may have received vaccinations at other institutions without those data being entered into the national vaccine registry. Third, although we performed adjusted analyses controlling for age and sex, this study remained primarily descriptive, and residual confounding due to underlying disease categories, treatment heterogeneity, and other unmeasured factors could not be excluded. Finally, although the coronavirus disease 2019 (COVID-19) pandemic had effectively ended and influenza activity had largely returned to patterns similar to those of the pre-pandemic period at the time of the survey, there was minimal influenza circulation during the 2020–2022 seasons [28]. Therefore, awareness of influenza vaccination may have declined substantially among both the public and healthcare professionals. This decline may have contributed to the low influenza vaccination rate. Nevertheless, a major strength of this study is that it was conducted at the largest rheumatology center in Korea, which enabled the inclusion of many patients with NRRDs, a sample size that would have been difficult to achieve at other institutions. This strengthens the robustness of the findings, despite the aforementioned limitations.

In conclusion, vaccination coverage among adults with NRRDs was low, particularly among those in the HLI group. Systematic vaccination strategies are needed to improve protection against VPDs in this high-risk population.

## Figures and Tables

**Figure f2-epih-48-e2026018:**
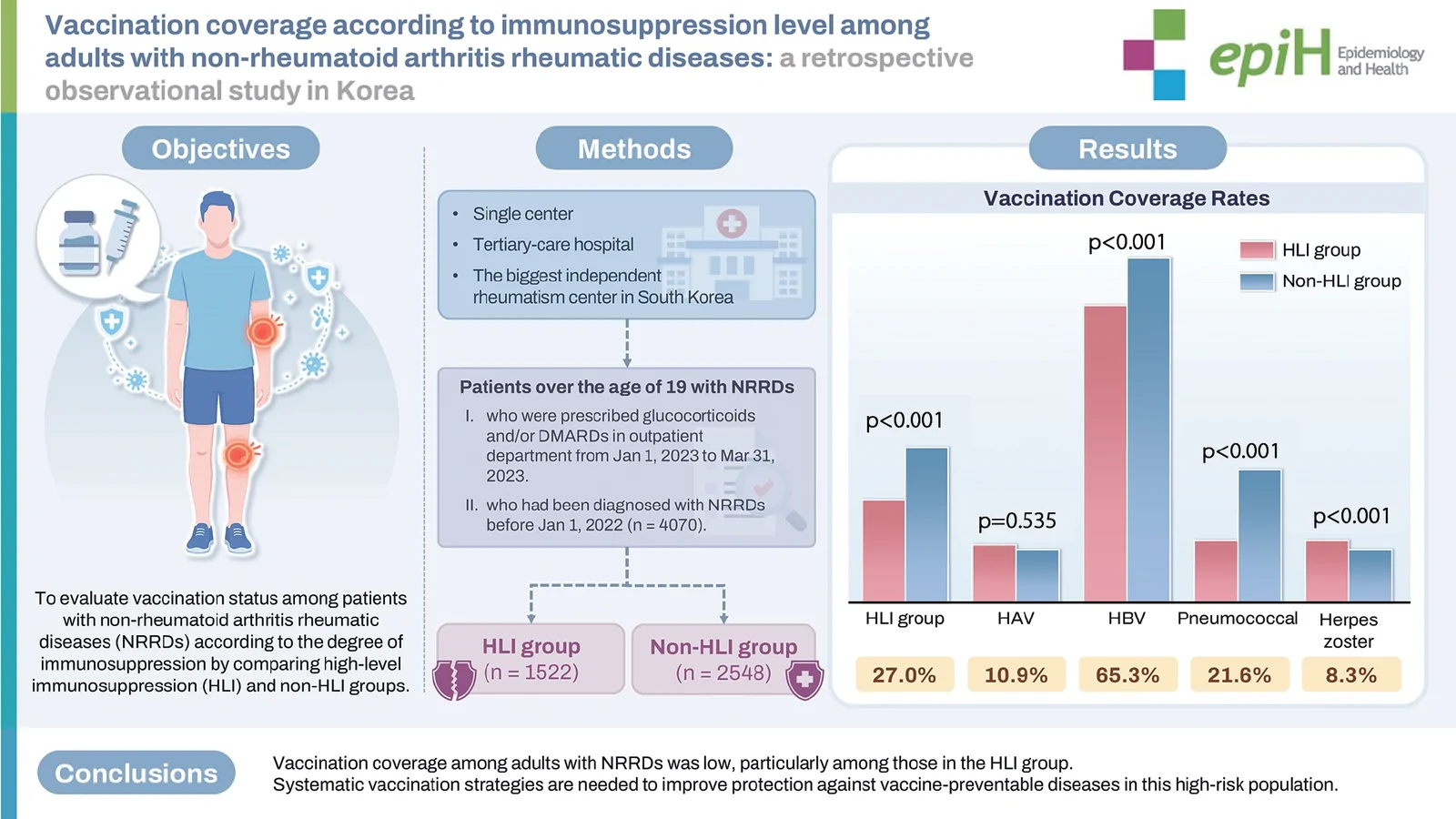


**Table 1.** Comparison of demographic characteristics between the HLI and non-HLI groups

Characteristics	Total (n=4,070)	HLI group (n=1,522)	Non-HLI group (n=2,548)	p-value^1^
Age, mean±SD (yr)	45.3±13.7	42.6±12.4	47.0±14.2	<0.001
Female sex	2,614 (64.2)	419 (27.5)	2,195 (86.1)	<0.001
Underlying non-RA rheumatic disease				
Systemic lupus erythematosus	1,588 (39.0)	80 (5.3)	1,508 (59.2)	<0.001
Ankylosing spondylitis	1,571 (38.6)	1,334 (87.6)	237 (9.3)	<0.001
Other systemic connective tissue diseases	757 (18.6)	68 (4.5)	689 (27.0)	<0.001
Dermatomyositis	160 (3.9)	3 (0.2)	157 (6.2)	<0.001
Juvenile idiopathic arthritis	109 (2.7)	69 (4.5)	40 (1.6)	<0.001
Systemic sclerosis	93 (2.3)	5 (0.3)	88 (3.5)	<0.001
Adult-onset Still disease	75 (1.8)	23 (1.5)	52 (2.0)	0.232
Polyarteritis nodosa and related conditions	11 (0.3)	1 (0.1)	10 (0.4)	0.062
Other necrotizing vasculopathies	6 (0.1)	2 (0.1)	4 (0.2)	1.000
Microscopic polyangiitis	0 (0)	0 (0)	0 (0)	-
Antiphospholipid syndrome	0 (0)	0 (0)	0 (0)	-
Other medical conditions				
Asplenia	11 (0.3)	1 (0.1)	10 (0.4)	0.062
Solid organ transplantation	3 (0.1)	0 (0)	3 (0.1)	0.297
Hematopoietic stem cell transplantation	0 (0)	0 (0)	0 (0)	-

Values are presented as number (%). HLI, high-level immunosuppression; SD, standard deviation; RA, rheumatoid arthritis.

1 Comparison between the HLI and non-HLI groups.

**Table 2.** Types of medications used in the HLI and non-HLI groups

Type of medication	HLI group (n=1,522)	Non-HLI group (n=2,548)
bDMARDs		
Adalimumab (TNF-α inhibitor)	487 (32.0)	0 (0)
Etanercept (TNF-α inhibitor)	459 (30.2)	0 (0)
Infliximab (TNF-α inhibitor)	270 (17.7)	0 (0)
Golimumab (TNF-α inhibitor)	182 (12.0)	0 (0)
Certolizumab (TNF-α inhibitor)	0 (0)	0 (0)
Tocilizumab (IL-6 inhibitor)	41 (2.7)	0 (0)
Belimumab (BLyS inhibitor)	34 (2.2)	0 (0)
Abatacept (CD80 and CD86 inhibitor)	22 (1.5)	0 (0)
Anakinra (IL-1 inhibitor)	0 (0)	0 (0)
Secukinumab (IL-17A inhibitor)	0 (0)	0 (0)
Rituximab (CD20 inhibitor)	0 (0)	0 (0)
tsDMARDs		
Baricitinib (JAK inhibitor)	0 (0)	0 (0)
Tofacitinib (JAK inhibitor)	0 (0)	0 (0)
csDMARDs		
Hydroxychloroquine	65 (4.3)	1,825 (71.6)
Methotrexate (≤0.4 mg/kg/wk)	108 (7.1)	558 (21.9)
Sulfasalazine	28 (1.8)	259 (10.2)
Azathioprine (≤3 mg/kg/day)	7 (0.5)	165 (6.5)
Cyclosporine	11 (0.7)	158 (6.2)
Leflunomide	8 (0.5)	121 (4.8)
D-penicillamine	0 (0)	4 (0.2)
Cyclophosphamide	0 (0)	1 (0.0)
Tacrolimus	0 (0)	0 (0)
Corticosteroids		
Prednisolone or equivalent, high dose (≥20 mg/day and ≥14 day)	35 (2.3)	0 (0)
Prednisolone or equivalent, low dose (<20 mg/day or <14 day)	0 (0)	1,678 (65.9)

Values are presented as number (%). HLI, high-level immunosuppression; DMARDs, disease-modifying antirheumatic drugs; bDMARDs, biologic DMARDs; tsDMARDs, targeted synthetic DMARDs; csDMARDs, conventional synthetic DMARDs; TNF, tumor necrosis factor; IL, interleukin; CD, cluster of differentiation; JAK, Janus kinase.

**Table 3.** Vaccination coverage in the HLI and non-HLI groups

Vaccination coverage	Total (n=4,070)	HLI group (n=1,522)				Non-HLI group (n=2,548)				Risk difference (95% CI)	p-value^1^	Absolute SMD
	Completely+Partially vaccinated	Completely vaccinated	Partially vaccinated	Unvaccinated	Not indicated	Completely vaccinated	Partially vaccinated	Unvaccinated	Not indicated			
Influenza^2^	1,098 (27.0)	308 (20.2)	-	1,214 (79.8)	-	790 (31.0)	-	1,758 (69.0)	-	−0.11 (−0.14, −0.08)	<0.001	0.249
HAV^3^	444 (10.9)	95 (6.2)	77 (5.1)	579 (38.0)	771 (50.7)	162 (6.2)	110 (4.3)	670 (26.3)	1,606 (63.0)	0.01 (−0.01, 0.03)	0.535	0.020
HBV^4^	2,659 (65.3)	841 (55.3)	64 (4.2)	609 (40.0)	8 (0.5)	1,672 (65.6)	82 (3.2)	791 (31.0)	3 (0.1)	−0.09 (−0.12, −0.06)	<0.001	0.196
Tdap/Td^5^	659 (16.2)	245 (16.1)	-	1,277 (83.9)	-	414 (16.2)	-	2,134 (83.8)	-	−0.00 (−0.03, 0.02)	0.899	0.004
Pneumococcal^6^	881 (21.6)	27 (1.8)	169 (11.1)	1,326 (87.1)	-	95 (3.7)	590 (23.2)	1,863 (73.1)	-	−0.14 (−0.16, −0.12)	<0.001	0.356
Herpes zoster^7^	341 (8.3)	63 (4.1)	4 (0.2)	1,436 (94.4)	19 (1.3)	265 (10.4)	9 (0.4)	2,211 (86.8)	63 (2.5)	−0.06 (−0.08, −0.05)	<0.001	0.242
HPV^8^	61 (1.5)	4 (0.3)	2 (0.1)	129 (8.5)	1,387 (91.1)	33 (1.3)	22 (0.9)	136 (5.3)	2,357 (92.5)	−0.02 (−0.02, −0.01)	<0.001	0.158

Values are presented as number (%). HLI, high-level immunosuppression; CI, confidence interval; SMD, standardized mean difference; HAV, hepatitis A virus; HBV, hepatitis B virus; Tdap, tetanus/diphtheria/acellular pertussis; Td, tetanus/diphtheria; PCV13, 13-valent pneumococcal conjugate vaccine; PPSV23, 23-valent pneumococcal polysaccharide vaccine; HPV, human papillomavirus.

1 Comparison of the combined total of the completely and partially vaccinated participants between the HLI and non-HLI groups.

2 Influenza: “Completely vaccinated” was defined as receipt of influenza vaccination within 1 year.

3 HAV: The following criteria were applied for HAV vaccination status: Completely vaccinated: Individuals who had received 2 doses of vaccination; Partially vaccinated: Individuals who had received only 1 dose of vaccination; Unvaccinated: Individuals younger than 40 years who had received no vaccination and had no evidence of HAV antibody (Ab) positivity, or individuals aged 40 years or older with HAV Ab negativity and no vaccination history; Not indicated: Individuals with HAV Ab positivity and no vaccination history, or individuals aged 40 years or older with no HAV Ab laboratory information and no vaccination history.

4 HBV: The following criteria were applied for HBV vaccination status: Completely vaccinated: Individuals who had completed 3 doses of vaccination or had HBs Ab positivity; Partially vaccinated: Individuals who had received 1 or 2 doses of vaccination; Unvaccinated: Individuals who had received no vaccination; Not indicated: Individuals who were HBV carriers.

5 Tdap/Td: “Completely vaccinated” was defined as receipt of Tdap or Td vaccination within 10 years.

6 Pneumococcal: The following criteria were applied for pneumococcal vaccination status: Completely vaccinated: Individuals who had received both PCV13 and PPSV23; Partially vaccinated: Individuals who had received either PCV13 or PPSV23 only; Unvaccinated: Individuals who had received neither PCV13 nor PPSV23.

7 Herpes zoster (live/inactivated): The following criteria were applied for herpes zoster vaccination status: Completely vaccinated: Individuals aged 50 years or older who had received 1 dose of the live vaccine, or individuals who had received 2 doses of the inactivated (recombinant) vaccine regardless of age; Partially vaccinated: Individuals who had received only 1 dose of the inactivated vaccine; Unvaccinated: Individuals who had received neither the live nor the inactivated vaccine; Not indicated: Individuals younger than 50 years who had received the live vaccine.

8 HPV: The following criteria were applied for HPV vaccination status: Completely vaccinated: Individuals who had completed 3 doses of vaccination; Partially vaccinated: Individuals who had received 1 or 2 doses of vaccination; Unvaccinated: Individuals who had received no vaccination; Not indicated: Individuals older than 26 years.

**Table 4.** Multivariable logistic regression analysis of factors associated with vaccination coverage after adjustment for age and sex

Vaccination coverage	Total (n)	HLI group		Non-HLI group		aOR (95% CI) (ref: non-HLI)	p-value^1^
		Vaccinated	Unvaccinated	Vaccinated	Unvaccinated		
Influenza	4,070	308 (20.2)	1,214 (79.8)	790 (31.0)	1,758 (69.0)	0.85 (0.70, 1.03)	0.095
HAV	1,693	172 (22.9)	579 (77.1)	272 (28.9)	670 (71.1)	0.90 (0.67, 1.21)	0.490
HBV	4,059	905 (59.8)	609 (40.2)	1,754 (68.9)	791 (31.1)	0.77 (0.65, 0.91)	0.002
Tdap/Td	4,070	245 (16.1)	1,277 (83.9)	414 (16.2)	2,134 (83.8)	0.76 (0.60, 0.96)	0.019
Pneumococcal	4,070	196 (12.9)	1,326 (87.1)	685 (26.9)	1,863 (73.1)	0.71 (0.57, 0.89)	0.003
Herpes zoster	3,988	67 (4.5)	1,436 (95.5)	274 (11.0)	2,211 (89.0)	0.79 (0.56, 1.09)	0.154
HPV	326	6 (4.4)	129 (95.6)	55 (28.8)	136 (71.2)	0.48 (0.18, 1.28)	0.142

Values are presented as number (%). HLI, high-level immunosuppression; aOR, adjusted odds ratio; CI, confidence interval; ref, reference; HAV, hepatitis A virus; HBV, hepatitis B virus; Tdap, tetanus/diphtheria/acellular pertussis; Td, tetanus/diphtheria; HPV, human papillomavirus.

1 Comparison of the vaccinated group, defined as the combined total of the completely and partially vaccinated participants, between the HLI and non-HLI groups.

## Supplementary materials

Supplementary Material 1. List of ‘non-RA rheumatic diseases (NRRD)’ included in this study

Supplementary Material 2. List of Vaccine-Preventable Diseases (VPDs) in Adults

Supplementary Material 3. Types of disease modifying anti-rheumatic drugs (DMARDs)

Supplementary Material 4. Vaccination coverage in male patients of the high-level immunosuppression (HLI) and non-HLI groups

Supplementary Material 5. Vaccination coverage in female patients of the high-level immunosuppression (HLI) and non-HLI groups

Supplementary Material 6. Comparison of HAV vaccination rates between the HLI and non-HLI groups stratified by age (<40 vs ≥40 years)

Supplementary Material 7. Comparison of Peumococcal vaccination rates between the HLI and non-HLI groups stratified by age (<65 vs ≥65 years)

Supplementary Material 8. Comparison of Herpes zoster vaccination rates between the HLI and non-HLI groups stratified by age (<50 vs ≥50 years)
