# Supplementary material for: Vaccination coverage according to immunosuppression level among adults with non-rheumatoid arthritis rheumatic diseases: a retrospective observational study in Korea
Source: Epidemiol Health. 2026 Apr 23;48:e2026018. doi: 10.4178/epih.e2026018 (PMC13503067; doi:10.4178/epih.e2026018)
Supplement: Supplementary file 1 [file epih-48-e2026018-Supplementary-1.docx]

## Supplementary Material 1. List of ‘non-RA rheumatic diseases (NRRD)’ included in this study

Non-RA rheumatic diseases (NRRD)

Systemic lupus erythematosus (SLE)

Ankylosing spondylitis

Systemic sclerosis

Dermatomyositis / Polymyositis

Juvenile idiopathic arthritis

Adult-onset Still’s disease

Polyarteritis nodosa

Microscopic polyangiitis

Antiphospholipid syndrome

Other systemic connective tissue diseases

Other necrotizing vasculopathies
