# Supplementary material for: Vaccination coverage according to immunosuppression level among adults with non-rheumatoid arthritis rheumatic diseases: a retrospective observational study in Korea
Source: Epidemiol Health. 2026 Apr 23;48:e2026018. doi: 10.4178/epih.e2026018 (PMC13503067; doi:10.4178/epih.e2026018)
Supplement: Supplementary file 2 [file epih-48-e2026018-Supplementary-2.docx]

## Supplementary Material 2. List of Vaccine-Preventable Diseases (VPDs) in Adults

Vaccine-Preventable Disease

Influenza

Hepatitis A

Hepatitis B

Measles, Mumps, Rubella

Tetanus, Diphtheria, Pertussis

Pneumococcal disease

Meningococcal disease

Haemophilus influenzae type b

Varicella (Chickenpox)

Herpes zoster (Shingles)

Human papillomavirus (HPV) associated disease
