# Supplementary material for: Vaccination coverage according to immunosuppression level among adults with non-rheumatoid arthritis rheumatic diseases: a retrospective observational study in Korea
Source: Epidemiol Health. 2026 Apr 23;48:e2026018. doi: 10.4178/epih.e2026018 (PMC13503067; doi:10.4178/epih.e2026018)
Supplement: Supplementary file 3 [file epih-48-e2026018-Supplementary-3.docx]

## Supplementary Material 3. Types of disease modifying anti-rheumatic drugs (DMARDs)

| csDMARDs | bDMARDs | tsDMARDs |
| --- | --- | --- |
| Methotrexate | Adalimumab (TNF-α inhibitor) | Baricitinib (JAK inhibitor) |
| Hydroxychloroquine | Etanercept (TNF-α inhibitor) | Tofacitinib (JAK inhibitor) |
| Sulfasalazine | Infliximab (TNF-α inhibitor) |  |
| Leflunomide | Golimumab (TNF-α inhibitor) |  |
| Azathioprine | Certolizumab (TNF-α inhibitor) |  |
| Cyclosporine | Tocilizumab (IL-6 inhibitor) |  |
| Tacrolimus | Belimumab (BLyS inhibitor) |  |
| Cyclophosphamide | Abatacept (CTLA-4 inhibitor) |  |
| D-penicillamine | Anakinra (IL-1 inhibitor) |  |
|  | Rituximab (CD20 inhibitor) |  |
|  | Secukinumab (IL-17 inhibitor) |  |
