# Supplementary material for: Vaccination coverage according to immunosuppression level among adults with non-rheumatoid arthritis rheumatic diseases: a retrospective observational study in Korea
Source: Epidemiol Health. 2026 Apr 23;48:e2026018. doi: 10.4178/epih.e2026018 (PMC13503067; doi:10.4178/epih.e2026018)
Supplement: Supplementary file 4 [file epih-48-e2026018-Supplementary-4.docx]

**Supplementary Material 4.** Vaccination coverage in male patients of the high-level immunosuppression (HLI) and non-HLI groups

|  | Total  (n = 1456) | HLI group  (n = 1103) | | | | Non-HLI group  (n = 353) | | | | Risk Difference (95% CI) | *P*^†^ | Absolute SMD |
| --- | --- | --- | --- | --- | --- | --- | --- | --- | --- | --- | --- | --- |
| Vaccination coverage | Completely + Partially  vaccinated | Completely vaccinated | Partially  vaccinated | Unvaccinated | Not  indicated | Completely vaccinated | Partially  vaccinated | Unvaccinated | Not  indicated |  |  |  |
| Influenza^a^ | 284 (19.5) | 195 (17.7) | - | 908 (82.3) | - | 89 (25.2) | - | 264 (74.8) | - | -0.075 (-0.126, -0.025) | 0.002 | 0.184 |
| HAV^b^ | 76 (5.2) | 66 (6.0) | 58 (5.3) | 449 (40.7) | 530 (48.1) | 10 (2.8) | 22 (6.2) | 118 (33.4) | 203 (57.5) | 0.022 (-0.014, 0.057) | 0.25 | 0.072 |
| HBV^c^ | 815 (56.0) | 595 (54.0) | 47 (4.3) | 453 (41.1) | 8 (0.7) | 220 (62.3) | 8 (2.3) | 124 (35.1) | 1 (0.3) | -0.064 (-0.122, -0.006) | 0.033 | 0.131 |
| Tdap/Td^d^ | 248 (17.0) | 184 (16.7) | - | 919 (83.3) | - | 64 (18.1) | - | 289 (81.9) | - | -0.014 (-0.060, 0.031) | 0.529 | 0.038 |
| Pneumococcal^e^ | 18 (1.2) | 12 (1.1) | 89 (8.1) | 1002 (90.8) | - | 6 (1.7) | 70 (19.8) | 277 (78.5) | - | -0.124 (-0.170, -0.078) | <0.001 | 0.348 |
| Herpes zoster^f^ | 55 (3.8) | 34 (3.1) | 1 (0.1) | 1056 (95.7) | 12 (1.1) | 21 (5.9) | 1 (0.3) | 329 (93.2) | 2 (0.6) | -0.031 (-0.058, -0.003) | 0.01 | 0.145 |
| HPV^g^ | 0 | 0 | 0 | 109 (9.9) | 994 (90.1) | 0 | 1 (0.3) | 39 (11.1) | 313 (88.7) | -0.003 (-0.008, 0.003) | 0.242 | 0.075 |

Values are presented as number (%).

HLI, High-level immunosuppression; CI, confidence interval; SMD, standerdized mean difference; HAV, Hepatitis A virus; HBV, Hepatitis B virus; Tdap, Tetanus/Diphtheria/Pertussis; Td, Tetanus/Diphtheria; HPV, Human papilloma virus.

^a^Influenza: ‘Completely vaccinated’ was defined as individuals who received influenza vaccination within 1 year.

^b^HAV: The following criteria were applied for HAV vaccination status.

- Completely vaccinated: Defined as individuals who received two doses of vaccination.

- Partially vaccinated: Defined as individuals who received only one dose of vaccination.

- Unvaccinated: Defined as individuals younger than 40 years who never received vaccination and had no evidence of HAV Ab (+), or individuals aged 40 years or older with HAV Ab (-) and no vaccination history.

- Not indicated: Defined as individuals with HAV Ab (+) and no vaccination history, or individuals aged 40 years or older without laboratory information of HAV Ab and no vaccination history.

^c^HBV: The following criteria were applied for HBV vaccination status.

- Completely vaccinated: Defined as individuals who completed three doses of vaccination or those with HBs Ab (+).

- Partially vaccinated: Defined as individuals who received one or two doses of vaccination.

- Unvaccinated: Defined as individuals who never received vaccination.

- Not indicated: Defined as individuals who were HBV carriers.

^d^Tdap/Td: ‘Completely vaccinated’ was defined as individuals who received Tdap or Td vaccination within 10 years.

^e^Pneumococcal: The following criteria were applied for pneumococcal vaccination status.

- Completely vaccinated: Defined as individuals who received both PCV13 and PPSV23 vaccinations.

- Partially vaccinated: Defined as individuals who received either PCV13 or PPSV23 vaccination only.

- Unvaccinated: Defined as individuals who received neither PCV13 nor PPSV23 vaccination.

^f^Herpes zoster (Live/Inactivated): The following criteria were applied for herpes zoster vaccination status.

- Completely vaccinated: Defined as individuals aged 50 years or older who received one dose of the live vaccine, or individuals who received two doses of the inactivated (recombinant) vaccine regardless of age.

- Partially vaccinated: Defined as individuals who received only one dose of the inactivated (recombinant) vaccine.

- Unvaccinated: Defined as individuals who received neither the live nor inactivated vaccine.

- Not indicated: Defined as individuals younger than 50 years who received live vaccine.

^g^HPV: The following criteria were applied for HPV vaccination status.

- Completely vaccinated: Defined as individuals who completed three doses of vaccination.

- Partially vaccinated: Defined as individuals who received one or two doses of vaccination.

- Unvaccinated: Defined as individuals who never received vacciantion.

- Not indicated: Defined as individuals older than 26 years.

^†^Comparison of the combined total of the completely and partially vaccinated groups between the HLI and non-HLI groups.
