# Supplementary material for: Vaccination coverage according to immunosuppression level among adults with non-rheumatoid arthritis rheumatic diseases: a retrospective observational study in Korea
Source: Epidemiol Health. 2026 Apr 23;48:e2026018. doi: 10.4178/epih.e2026018 (PMC13503067; doi:10.4178/epih.e2026018)
Supplement: Supplementary file 5 [file epih-48-e2026018-Supplementary-5.docx]

**Supplementary Material 5.** Vaccination coverage in female patients of the high-level immunosuppression (HLI) and non-HLI groups

|  | Total  (n = 2614) | HLI group  (n = 419) | | | | Non-HLI group  (n = 2195) | | | | Risk Difference (95% CI) | *P*^†^ | Absolute SMD |
| --- | --- | --- | --- | --- | --- | --- | --- | --- | --- | --- | --- | --- |
| Vaccination coverage | Completely + Partially  vaccinated | Completely vaccinated | Partially  vaccinated | Unvaccinated | Not  indicated | Completely vaccinated | Partially  vaccinated | Unvaccinated | Not  indicated |  |  |  |
| Influenza^a^ | 814 (31.1) | 113 (27.0) | - | 306 (73.0) | - | 701 (31.9) | - | 1494 (68.1) | - | -0.050 (-0.096, -0.003) | 0.044 | 0.109 |
| HAV^b^ | 178 (6.8) | 29 (6.9) | 19 (4.5) | 130 (31.0) | 241 (57.5) | 152 (6.9) | 88 (4.0) | 552 (25.2) | 1403 (63.9) | 0.005 (-0.028, 0.038) | 0.755 | 0.017 |
| HBV^c^ | 1698 (65.0) | 246 (58.7) | 17 (4.1) | 156 (37.2) | 0 | 1452 (66.2) | 74 (3.4) | 667 (30.4) | 2 (0.1) | -0.068 (-0.118, -0.017) | 0.006 | 0.143 |
| Tdap/Td^d^ | 411 (15.7) | 61 (14.6) | - | 358 (85.4) | - | 350 (15.9) | - | 1845 (84.1) | - | -0.014 (-0.051, 0.023) | 0.475 | 0.039 |
| Pneumococcal^e^ | 104 (4.0) | 15 (3.6) | 80 (19.1) | 324 (77.3) | - | 89 (4.1) | 520 (23.7) | 1586 (72.3) | - | -0.051 (-0.095, -0.006) | 0.032 | 0.117 |
| Herpes zoster^f^ | 273 (10.4) | 29 (6.9) | 3 (0.7) | 380 (90.7) | 7 (1.7) | 244 (11.1) | 8 (0.4) | 1882 (85.7) | 61 (2.8) | -0.038 (-0.067, -0.010) | 0.021 | 0.131 |
| HPV^g^ | 37 (1.4) | 4 (1.0) | 2 (0.5) | 20 (4.8) | 393 (93.8) | 33 (1.5) | 21 (1.0) | 97 (4.4) | 2044 (93.1) | -0.010 (-0.023, 0.003) | 0.198 | 0.074 |

Values are presented as number (%).

HLI, High-level immunosuppression; CI, confidence interval; SMD, standerdized mean difference; HAV, Hepatitis A virus; HBV, Hepatitis B virus; Tdap, Tetanus/Diphtheria/Pertussis; Td, Tetanus/Diphtheria; HPV, Human papilloma virus.

^a^Influenza: ‘Completely vaccinated’ was defined as individuals who received influenza vaccination within 1 year.

^b^HAV: The following criteria were applied for HAV vaccination status.

- Completely vaccinated: Defined as individuals who received two doses of vaccination.

- Partially vaccinated: Defined as individuals who received only one dose of vaccination.

- Unvaccinated: Defined as individuals younger than 40 years who never received vaccination and had no evidence of HAV Ab (+), or individuals aged 40 years or older with HAV Ab (-) and no vaccination history.

- Not indicated: Defined as individuals with HAV Ab (+) and no vaccination history, or individuals aged 40 years or older without laboratory information of HAV Ab and no vaccination history.

^c^HBV: The following criteria were applied for HBV vaccination status.

- Completely vaccinated: Defined as individuals who completed three doses of vaccination or those with HBs Ab (+).

- Partially vaccinated: Defined as individuals who received one or two doses of vaccination.

- Unvaccinated: Defined as individuals who never received vaccination.

- Not indicated: Defined as individuals who were HBV carriers.

^d^Tdap/Td: ‘Completely vaccinated’ was defined as individuals who received Tdap or Td vaccination within 10 years.

^e^Pneumococcal: The following criteria were applied for pneumococcal vaccination status.

- Completely vaccinated: Defined as individuals who received both PCV13 and PPSV23 vaccinations.

- Partially vaccinated: Defined as individuals who received either PCV13 or PPSV23 vaccination only.

- Unvaccinated: Defined as individuals who received neither PCV13 nor PPSV23 vaccination.

^f^Herpes zoster (Live/Inactivated): The following criteria were applied for herpes zoster vaccination status.

- Completely vaccinated: Defined as individuals aged 50 years or older who received one dose of the live vaccine, or individuals who received two doses of the inactivated (recombinant) vaccine regardless of age.

- Partially vaccinated: Defined as individuals who received only one dose of the inactivated (recombinant) vaccine.

- Unvaccinated: Defined as individuals who received neither the live nor inactivated vaccine.

- Not indicated: Defined as individuals younger than 50 years who received live vaccine.

^g^HPV: The following criteria were applied for HPV vaccination status.

- Completely vaccinated: Defined as individuals who completed three doses of vaccination.

- Partially vaccinated: Defined as individuals who received one or two doses of vaccination.

- Unvaccinated: Defined as individuals who never received vacciantion.

- Not indicated: Defined as individuals older than 26 years.

^†^Comparison of the combined total of the completely and partially vaccinated groups between the HLI and non-HLI groups.
