# Supplementary material for: Vaccination coverage according to immunosuppression level among adults with non-rheumatoid arthritis rheumatic diseases: a retrospective observational study in Korea
Source: Epidemiol Health. 2026 Apr 23;48:e2026018. doi: 10.4178/epih.e2026018 (PMC13503067; doi:10.4178/epih.e2026018)
Supplement: Supplementary file 6 [file epih-48-e2026018-Supplementary-6.docx]

|  | HAV (Age < 40) (n = 1481) | |  |  |  |
| --- | --- | --- | --- | --- | --- |
|  | HLI Group (n = 658) | Non-HLI Group (n = 823) | *P*-value | RD (95% CI) | \|SMD\| |
| Complete Vaccinated | 48 (7.3%) | 101 (12.3%) | 0.003 | -0.063 (-0.102, -0.024) | 0.163 |
| Partial Vaccinated | 50 (7.6%) | 71 (8.6%) |  |  |  |
| Unvaccinated | 558 (84.8%) | 639 (77.6%) |  |  |  |
| Not Indicated | 2 (0.3%) | 12 (1.5%) |  |  |  |
|  | | | | | |
|  | HAV (Age ≥ 40) (n = 2589) | |  |  |  |
|  | HLI Group (n = 864) | Non-HLI Group (n = 1725) | *P*-value | RD (95% CI) | \|SMD\| |
| Complete Vaccinated | 47 (5.4%) | 61 (3.5%) | 0.909 | 0.016 (-0.095, 0.126) | 0.037 |
| Partial Vaccinated | 27 (3.1%) | 39 (2.3%) |  |  |  |
| Unvaccinated | 21 (2.4%) | 31 (1.8%) |  |  |  |
| Not Indicated | 769 (89.0%) | 1594 (92.4%) |  |  |  |

**Supplementary Material 6.** Comparison of HAV vaccination rates between the HLI and non-HLI groups stratified by age (<40 vs ≥40 years)
