# Supplementary material for: Vaccination coverage according to immunosuppression level among adults with non-rheumatoid arthritis rheumatic diseases: a retrospective observational study in Korea
Source: Epidemiol Health. 2026 Apr 23;48:e2026018. doi: 10.4178/epih.e2026018 (PMC13503067; doi:10.4178/epih.e2026018)
Supplement: Supplementary file 7 [file epih-48-e2026018-Supplementary-7.docx]

|  | Pneumococcus (Age < 65) (n = 3678) | |  |  |  |
| --- | --- | --- | --- | --- | --- |
|  | HLI Group (n = 1436) | Non-HLI Group (n = 2242) | *P*-value | RD (95% CI) | \|SMD\| |
| Complete Vaccinated | 9 (0.6%) | 37 (1.7%) | < 0.001 | -0.107 (-0.129, -0.086) | 0.314 |
| Partial Vaccinated | 113 (7.9%) | 394 (17.6%) |  |  |  |
| Unvaccinated | 1314 (91.5%) | 1811 (80.8%) |  |  |  |
| Not Indicated | 0 (0.0%) | 0 (0.0%) |  |  |  |
|  | | | | | |
|  | Pneumococcus (Age >= 65) (n = 392) | |  |  |  |
|  | HLI Group (n = 86) | Non-HLI Group (n = 306) | *P*-value | RD (95% CI) | \|SMD\| |
| Complete Vaccinated | 18 (20.9%) | 58 (19.0%) | 0.611 | 0.030 (-0.054, 0.115) | 0.084 |
| Partial Vaccinated | 56 (65.1%) | 196 (64.1%) |  |  |  |
| Unvaccinated | 12 (14.0%) | 52 (17.0%) |  |  |  |
| Not Indicated | 0 (0.0%) | 0 (0.0%) |  |  |  |

**Supplementary Material 7.** Comparison of Peumococcal vaccination rates between the HLI and non-HLI groups stratified by age (<65 vs ≥65 years)
