# Supplementary material for: Vaccination coverage according to immunosuppression level among adults with non-rheumatoid arthritis rheumatic diseases: a retrospective observational study in Korea
Source: Epidemiol Health. 2026 Apr 23;48:e2026018. doi: 10.4178/epih.e2026018 (PMC13503067; doi:10.4178/epih.e2026018)
Supplement: Supplementary file 8 [file epih-48-e2026018-Supplementary-8.docx]

|  | Herpes zoster (Age < 50) (n = 2582) | |  |  |  |
| --- | --- | --- | --- | --- | --- |
|  | HLI Group (n = 1117) | Non-HLI Group (n = 1465) | *P*-value | RD (95% CI) | \|SMD\| |
| Complete Vaccinated | 1 (0.1%) | 2 (0.1%) | 0.156 | -0.005 (-0.009, 0.000) | 0.072 |
| Partial Vaccinated | 1 (0.1%) | 7 (0.5%) |  |  |  |
| Unvaccinated | 1096 (98.1%) | 1393 (95.1%) |  |  |  |
| Not Indicated | 19 (1.7%) | 63 (4.3%) |  |  |  |
|  | | | | | |
|  | Herpes zoster (Age ≥ 50) (n = 1488) | |  |  |  |
|  | HLI Group (n = 405) | Non-HLI Group (n = 1083) | *P*-value | RD (95% CI) | \|SMD\| |
| Complete Vaccinated | 62 (15.3%) | 263 (24.3%) | 0.001 | -0.084 (-0.128, -0.040) | 0.211 |
| Partial Vaccinated | 3 (0.7%) | 2 (0.2%) |  |  |  |
| Unvaccinated | 340 (84.0%) | 818 (75.5%) |  |  |  |
| Not Indicated | 0 (0.0%) | 0 (0.0%) |  |  |  |

**Supplementary Material 8.** Comparison of Herpes zoster vaccination rates between the HLI and non-HLI groups stratified by age (<50 vs ≥50 years)
